# Correlation between subsets of tumor-infiltrating immune cells and risk stratification in patients with cervical cancer

**DOI:** 10.7717/peerj.7804

**Published:** 2019-10-11

**Authors:** Rui Chen, Yi Gong, Dongling Zou, Lifeng Wang, Li Yuan, Qi Zhou

**Affiliations:** 1 Department of Pathology, Chongqing University Cancer Hospital & Chongqing Cancer Institute & Chongqing Cancer Hospital, Chongqing, China; 2 Department of Hematology-Oncology, Chongqing University Cancer Hospital & Chongqing Cancer Institute & Chongqing Cancer Hospital, Chongqing, China; 3 Department of Gynecologic Oncology, Chongqing University Cancer Hospital & Chongqing Cancer Institute & Chongqing Cancer Hospital, Chongqing, China

**Keywords:** Cervical cancer, CD45RO, Tumor microenvironment, FOXP3, Risk stratification, Tumor infiltrating T lymphocytes

## Abstract

**Aim:**

To investigate the correlation between clinicopathological features and risk stratification in cervical cancer patients, and evaluate the feasibility of tumor-infiltrating immune cells as prognostic biomarkers in clinical practice.

**Methods:**

CD3+ tumor infiltrating T cells (TILs), CD45RO+ TILs, CD4+ TILs, CD8+ TILs, FOXP3+ TILs (regulatory T cells, Tregs), CD68+ tumor associated macrophages (TAMs), CD163+ TAMs, and PD-L1+ tumor cells were immunostained in formalin-fixed paraffin-embedded (PPFE) tissues from 96 cervical cancer patients. Immunostaining density and other clinicopathological features such as age, FIGO stage, histopathologic type, Ki67 index, HPV status, lymhovasular invasion status (LVI), lymph node metastasis, tumor size, stromal invasion status, surgical margin status, and parametrial invasion, were evaluated for their roles in risk stratification of cervical cancer patients.

**Results:**

The results showed that significant differences of lymph node metastasis (*p* = 0.003), surgical margin status (*p* = 0.020), and stromal invasion status (*p* = 0.004) existed between lVI(−) and LVI(+) patients. CD3+ TILs in the central tumor area (*p* = 0.010), CD4+ TILs in the central tumor area (*p* = 0.045), CD8 + TILs in the central tumor area (*p* = 0.033), and CD8+ TILs in the invasive margin area (*p* = 0.004) showed significant differences between lVI(−) and LVI(+) patients. When patients were grouped by status of lymph node metastasis, significant differences of FIGO stage (*p* = 0.005), LVI status (*p* = 0.003), CD3+ TILs in the central tumor area (*p* = 0.045), CD45RO+ TILs in the central tumor area (*p* = 0.033), and CD45RO+ TILs in the invasive margin area (*p* = 0.028) were also observed. After the patients were stratified into low-, intermediate-, and high risk groups, significant differences of FIGO stage (*p* = 0.018), status of lymph node metastasis (*p* = 0.000), LVI status (*p* = 0.000), parametrial invasion status (p=0.012), stromal invasion status (*p* = 0.000), tumor growth pattern (*p* = 0.015) and tumor size (*p* = 0.000) were identified among 3 groups of patients, while only CD45RO+ TILs in the invasive margin area (*p* = 0.018) and FOXP3+ TILs in the central tumor area (*p* = 0.009) were statistically different among three groups of patients. Spearman’s correlation analysis demonstrated that FIGO stage, LVI status, status of lymph node metastasis, parametrial invasion, stromal invasion status, and tumor size positively correlated with risk stratification (*P* = 0.005, 0.020, 0.000, 0.022, 0.000, and 0.000 respectively), while CD45RO+ TILs in the invasive margin area and FOXP3+ TILs in the central tumor area showed statistically negative correlation with risk stratification (*P* = 0.031, 0.009 respectively).

**Conclusion:**

Our study suggested that CD45RO+ TILs in the invasive margin area and FOXP3+ TILs in the central tumor area might be useful biomarkers for risk stratification in cervical cancer patients. Large cohort studies of cervical cancer patients are required to validate our hypothesis.

## Introduction

Cervical cancer is one the most prevalent malignant diseases affecting women worldwide (Siegel, Miller & Jemal, 2019). Persistent chronic infection with high-risk human papillomavirus (HPV) such as HPV-16 and HPV-18 is the main cause of cervical cancer and its precursor lesions (Cohen et al., 2019; Torre et al., 2017). Despite great progress in tumor prevention, screening and treatment in recent years, cervical cancer is still one of the major reasons of morbidity and mortality among women in developing countries (Bhatla et al., 2018).

The interactions between tumor and immune system are critical for tumor initiation, progression and metastasis. Immune cells including lymphocytes, macrophages, neutrophils, mast cells, myeloid-derived suppressor cells, dendritic cells and natural killer cells were recruited into the tumor tissue as well as cytokines, fibroblasts and vasculatures, which made up a complex network of tumor microenvironment (Gajewski, Schreiber & Fu, 2013; Hanahan & Weinberg, 2011). The immune/inflammatory tumor microenvironment played important roles in tumor pathobiology, it was also associated with clinical outcome of various malignant diseases such as melanoma, breast cancer, lung cancer, colorectal cancer, and hematological malignancies (Becht et al., 2016; Quail & Joyce, 2013). Tumor infiltrating lymphocytes (TILs) and tumor associated macrophages (TAMs) as the main components of tumor microenvironment immune cells, has been reported as important biomarkers in predicting tumor prognosis and response to immunotherapy, and a new scoring system describing the intra-tumoral immune contexture including cell type, density and location of immune cells in tumor tissues has been proved to be reliable in estimate of recurrence risk for colon cancer patients, which supported the growing interests in utilization of immune/inflammatory tumor microenvironment features for risk stratification or novel immunotherapy for tumor patients (Goswami et al., 2017; Hendry et al., 2017a; Hendry et al., 2017b).

The HPV early 6 (E6) and early 7 (E7) gene encoded proteins are two well-known oncoproteins involved in the pathogenesis of cervical cancer, and defective T cell immunity against HPV has been considered an important microenvironment factor influencing tumor biological characteristics (Sheu et al., 2007; Van der Burg et al., 2007). Recently, the therapeutic value of adoptive transfer of TILs in HPV-associated epithelial cancers including cervical cancer has been reported (Stevanovic et al., 2019), and new immunotherapies such as Pembrolizumab, a humanized anti-PD-1 antibody, was approved by the US Food and Drug Administration for patients with recurrent or metastatic cervical cancer in 2018. However, the relationship between the tumor-infiltrative immune cells and clinicopathological features of cervical cancer has not been fully elucidated to date. In the present study, we aim to evaluate the distribution of tumor infiltrating T cells (TILs) and tumor associated macrophages (TAMs) in the inflammatory microenvironment of cervical cancer and to analyze the possible impacts on risk stratification of cervical cancer patients, which might provide new biomarkers for prognostication and prediction for response of immunotherapy in cervical cancer patients.

## Material and Methods

### Case selection

Ninety-six cases of cervical cancer diagnosed between 2014 and 2016 in Chongqing Cancer Institute/Hospital were included in the study based on the availability of complete clinical data and formalin-fixed, paraffin embedded (FFPE) tissues from tumors. All the cases were reviewed by two experienced pathologists according to the criteria of the fourth edition of WHO Classification of tumors of female reproductive organs. Clinicopathological parameters including age, FIGO staging, diagnosis, histological grade, Ki67 index, tumor size, lymph node status, lymphvascular invasion (LVI), parametrial invasion, surgical margin status, white blood cell (WBC) count, and imaging examinations (ultrasonic examination and radiologic examination of brain, chest, abdomen and pelvis) at the time of diagnosis were collected. SLAN-96P type fluorescence quantitative polymerase chain reaction (PCR) instrument, manufactured by Shanghai Hongshi Medical Technology Co., Ltd., was used for HPV genotyping in the study. The high risk HPV nucleic acid typing kit was provided by Shanghai ZJ Bio-Tech Co., Ltd. (15 HR-HPV subtypes consisting of HPV16, 18, 31, 33, 35, 39, 45, 51, 52, 56, 58, 59, 66, 68 and 82; the human single copy gene MNBH was used as an internal control). Blood samples were taken for biochemical tests of liver and kidney functions, which were performed by Hitachi Chemistry Analyzer 7600. The reagents for liver and kidney function assays were purchased from China Maccura Biotechnology Co., Ltd. The study was approved by the ethics committees of Chongqing University Cancer Hospital & Chongqing Cancer Institute & Chongqing Cancer Hospital (No. 2017-082).

### Immunohistochemical staining and analysis

Formalin-fixed, paraffin-embedded tissue samples contained cervical cancer and invasive margins were selected, nine tissue paraffin sections of 4 µm were processed for staining with primary monoclonal antibodies to anti-CD3 (clone SP35, rabbit monoclonal; Abcam, Cambridge, MA, USA), anti-CD45RO (clone SP35, rabbit monoclonal; Abcam, Cambridge, MA, USA), anti-CD4 (clone SP35, rabbit monoclonal; Abcam, Cambridge, MA, USA), anti-Foxp3 (clone 236A/E7, mouse monoclonal; Abcam), anti-CD8 (clone SP16, rabbit monoclonal; Abcam), anti-CD68 (clone KP-1, mouse monoclonal; Abcam), anti-CD163 (clone 10D6, mouse monoclonal; Abcam), and anti-PD-L1 (clone SP142, rabbit monoclonal; ORIGENE) using the GTVision III detection system (DAKO), according to the manufacturer’s instructions. Density of tumor-infiltrating immune cell subsets in the center (CT) and the invasive margin (IM) of cervical cancer were quantified as total counts of CD3, CD45RO, CD4, CD8, Foxp3, PD-L1, CD68, and CD163 positive cells per high power field (about 0.2 mm^2^) by manual inspection of stained sections with at least 10 fields of high staining intensity. The immune cells in the central area of tumor tissue (CT) and the immune cells surround the invasive margin (IM) of tumor tissue were recorded respectively. Membranous immunostaining for PD-L1 was considered as positive and scored by a staining intensity of tumor cells ranging from 0 to 3 (0 = no staining, 1 = weak staining, 2 = moderate staining, 3 = strong staining). All the immuno-staining sections were independently examined for technical and diagnostic qualities by 2 experienced pathologists in a blinded fashion.

### Statistical analysis

All data were analyzed with SPSS 18.0 (IBM Corporation, Armonk, NY, USA). Categorical variables were compared using chi-square test. The difference between continuous variables was assessed using *T* tests, ANOVA or Mann–Whitney *U* tests. *P* value of less than 0.05 was considered statistically significant. Spearman’s rank correlation coefficient analysis and Logistic regression analysis was performed to assess the association between risk stratification of cervical cancer patients and clinicopathological factors including the immunostaining results of microenvironment immune cells.

**Table 1:** Clinicopathological characteristics of patients with cervical cancer.

Characteristics	Number of cases (%)
Age (y)	
≥50	40 (41.7)
<50	56 (58.3)
FIGO stage	
≥IBII	49 (51.0)
<IBII	47 (49.0)
Histological type	
Squamous cell carcinoma	88 (91.7)
Adenocarcinoma	3 (3.1)
Adeno-squamous cell carcinoma	3 (3.1)
Neuroendocrine carcinoma	2 (2.1)
Tumor size (cm)	
Invisible lesion	5 (5.2)
<2	17 (17.7)
≥2, <4	59 (61.5)
≥4	15 (15.6)
Stromal invasion of uterine cervix	
Macroscopic growth pattern	
Exophytic nodular	65 (67.7)
Ulcerated nodular	5 (5.2)
Endophytic nodular	21 (21.9)
Flat lesion	5 (5.2)
Stromal invasion	
Superficial 1/3	36 (37.5)
Middle 1/3	15 (15.6)
Deep 1/3	45 (46.9)
LVI	
Yes	35 (36.5)
No	61 (63.5)
Lymph node metastasis	
Yes	14 (14.6)
No	82 (85.4)
Hr HPV status	
Negative	17 (21.0)
HPV-16 positive	47 (58.0)
HPV-18 positive	4 (4.9)
Other Hr HPV positive	13 (16.1)
Risk stratification	
Low risk	57 (59.4)
Intermediate risk	21 (21.9)
High risk	18 (18.7)

## Results

### Patient characteristics

There was 96 patients enrolled in this study, and the basic characteristics were depicted in Table 1. The median age was 48 years old (range 24–71 years) at the time of diagnosis. Sixty-four patients were in FIGO I stages, and 32 patients were in FIGO II stages, only one patient was FIGO III stages. Squamous cell carcinoma was the major histological type (88/96, 91.7%), while adenocarcinoma (3/96, 3.1%), adeno-squamous cell carcinoma (3/96, 3.1%) and neuroendocrine carcinoma (2/96, 2.1%) were less frequent in this study. The median Ki67 index of tumor cells was 0.80 (range 0.10–0.95). The median tumor size was 2.5 cm (range 1.0–7.0 cm). Exophytic nodular pattern (65/96, 67.7%) was the most frequent growth pattern of cervical cancer in this study, and endophytic nodular pattern (21/96, 21.9%) was the second most frequent growth pattern, while ulcerated nodular pattern (5/96, 5.2%) and flat lesion pattern (5/96, 5.2%) was less frequent in this study. LVI of tumor was detected in 35 cases (36.5%), and lymph node metastasis of tumor was detected in 14 cases (14.6%). Tumors with superficial 1/3 stromal invasion was observed in 36 cases (37.5%), and tumors with middle 1/3 stromal invasion was observed in 15 cases (15.6%), whereas tumors with deep 1/3 stromal invasion was observed in 45 cases (46.9%). Forty-seven (58.0%) patients carried HPV-16 infection, and 4 (4.9%) patients carried HPV-18 infection, while no high-risk HPV infection was detected in 17 (21.0%) patients. The patients were stratified into low-risk (57/96, 59.4%), intermediate-risk (21/96, 21.9%), and high risk (18/96, 18.7%) groups according to their prognostic factors (Bhatla et al., 2018; Cohen et al., 2019).

### Correlations between risk stratification and clinicopthological variables

**Figure 1:**
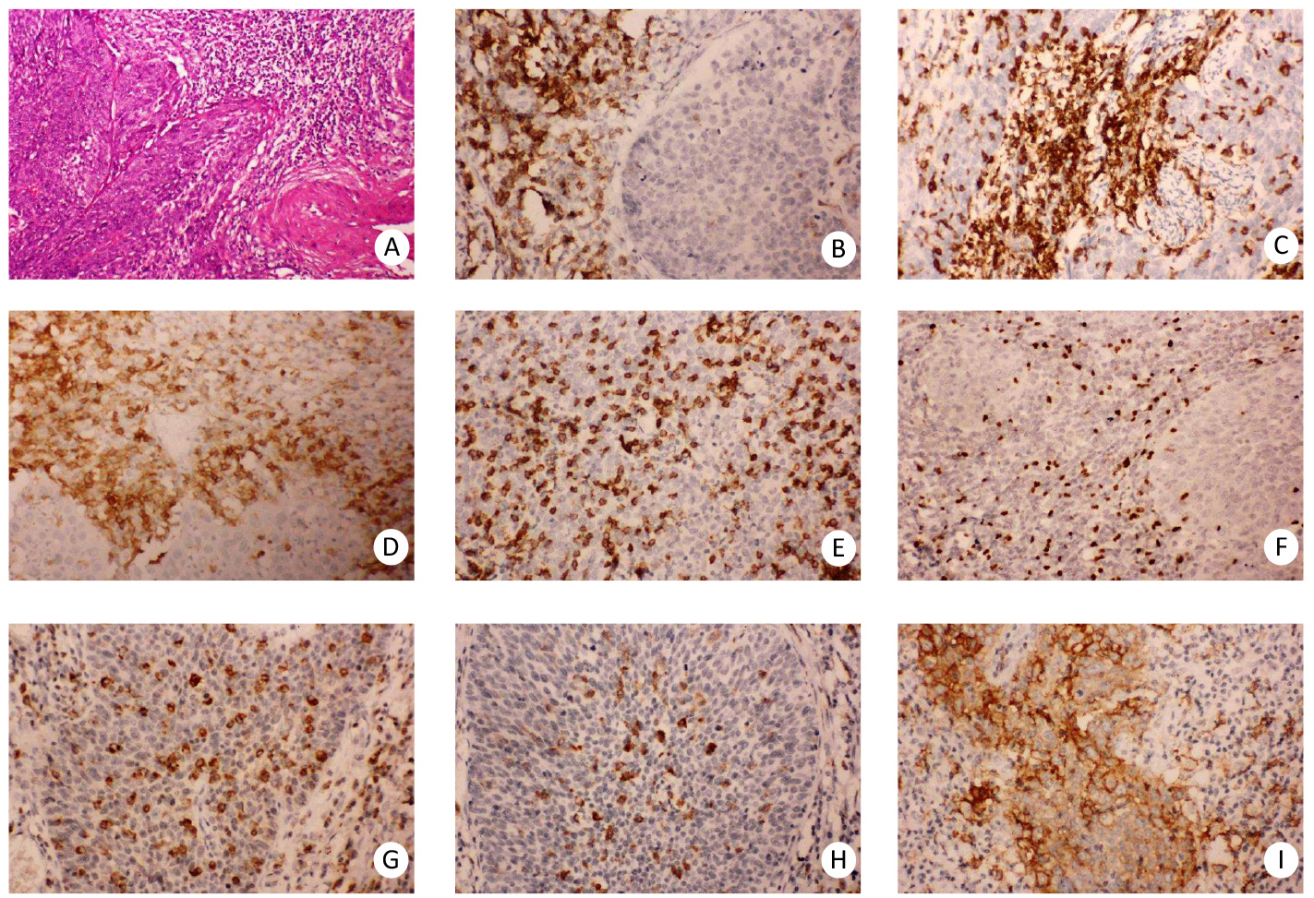
Infiltrating immune cells in the microenvironment of cervical cancer. Representative staining patterns of H.E (A), CD3 immunostaining (B), CD45RO immunostaining (C), CD4 immunostaining (D), CD8 immunostaining (E), FOXP3 immunostaining (F), CD68 immunostaining (G), CD163 immunostaining (H), PD-L1 immunostaining (I), Original magnification, ×100 (A), ×200 (B–I).

To investigate the relationship between risk stratification and clinicopthological features in cervical cancer patients, we examined the density of tumor infiltrating immune cells as well as the expression of PD-L1 in 96 cases of paraffin-embedded, formalin-fixed human cervical cancer tissues by immunohistochemistry staining. As shown in Fig. 1, immunostaining of CD45RO, CD4 demonstrated membrane positivity mainly in T lymphocytes, and immunostaining of CD3 and CD8 showed both membrane and cytoplasmic positivity mainly in T lymphocytes, while immunostaining of FOXP3 demonstrated nuclear positive pattern in certain subsets of regulatory T lymphocytes. Immunostaining of CD68 and CD163 demonstrated membrane and cytoplasmic positive pattern mainly in macrophages. Immunostaining of PD-L1 demonstrated membrane and cytoplasmic positive pattern mainly in tumor cells and macrophages.

**Table 2:** Comparison of clinicopathological features between cervical cancer patients in LVI positive and negative groups.

Variable	*n*	LVI (−)	LVI (+)	*P* value
***FIGO stage***	96			0.630
<IBII	47	31	16	
≥IBII	49	30	19	
**Histological type**	96			0.833
Squamous cell carcinoma	88	57	31	
adenocarcinoma	3	1	2	
Adenosquamous carcinoma	3	2	1	
Neuroendocrine small cell carcinoma	2	1	1	
**Lymph node metastasis**	96			0.003^**^
−	82	57	25	
+	14	4	10	
**Parametrial invasion**	96			0.059
−	94	61	33	
+	2	0	2	
**Surgical margin status**	96			0.020^*^
−	93	61	32	
+	3	0	3	
**Stromal invasion**	96			0.004^**^
Superficial 1/3	36	31	5	
Middle 1/3	15	8	7	
Deep 1/3	45	22	23	
**growth pattern**	96			0.507
Exophytic nodular	65	42	23	
Ulcerated nodular	5	4	1	
Endophytic nodular	21	11	10	
flat	5	4	1	
***Tumor size***	96			0.079
No visible lesion	5	4	1	
**<2**	17	14	3	
**≥2, <4**	59	37	22	
**≥4**	15	9	6	
***HPV status***	81			0.495
*Negative*	17	11	6	
*HPV-16* (+*)*	47	28	19	
*HPV-18* (+*)*	4	2	2	
*Other Hr HPV* (+*)*	13	11	2	
***Age (median(range))***	96	47(24–71)	48(27–63)	0.879
***Ki67 index*** ***(median(range))***	96	0.80(0.10–0.95)	0.75(0.20–0.90)	0.252
***CD3***_***IM***_***(median(range))***	96	220(40–500)	200(100–400)	0.115
***CD3***_***CT***_ ***(median(range))***	96	50(0–400)	20(0–200)	0.010^*^
***CD45RO***_***IM***_ ***(median(range))***	96	220(60–500)	200(100–350)	0.050
***CD45RO***_***CT***_ ***(median(range))***	96	40(0–350)	40(0–150)	0.114
***CD4***_***IM***_ ***(median(range))***	96	160(70–400)	160(50–350)	0.854
***CD4***_***CT***_ ***(median(range))***	96	20(0–180)	0(0–120)	0.045^*^
***CD8***_***IM***_ ***(median(range))***	96	180(30–400)	120(30–250)	0.004^**^
***CD8***_***CT***_ ***(median(range))***	96	50(0–400)	10(0–200)	0.033^*^
***FOXP3***_***IM***_ ***(median(range))***	96	100(0–350)	100(0–300)	0.876
***FOXP3***_***CT***_ ***(median(range))***	96	5(0–150)	0(0–50)	0.075
***CD68***_***IM***_ ***(median(range))***	96	100(0–210)	110(0–200)	0.608
***CD68***_***CT***_ ***(median(range))***	96	40(0–200)	30(0–110)	0.367
***CD163***_***IM***_ ***(median(range))***	96	100(0–220)	100(0–220)	0.522
***CD163***_***CT***_ ***(median(range))***	96	45(0–200)	30(0–160)	0.078
***PD-L1 expression***	96			0.553
-	45	26	19	
+	23	16	7	
++	19	14	5	
+++	9	5	4	

**Notes.**

* *P* < 0.05 ** *P* < 0.01

The clinicopathological features between patients grouped by status of LVI, lymph node metastasis, FIGO stages were statistically analyzed. According to Table 2, significant differences of lymph node metastasis (*p* = 0.003), surgical margin status (*p* = 0.020), stromal invasion status (*p* = 0.004) between lVI(-) and LVI(+) patients were observed. CD3+ TILs in the central tumor area (*p* = 0.010), CD4+ TILs in the central tumor area (*p* = 0.045), CD8+ TILs in the central tumor area (*p* = 0.033), and CD8+ TILs in the invasive margin area (*p* = 0.004) also showed significant difference between lVI(-) and LVI(+) patients. When the patients were grouped by status of lymph node, as shown in Table 3, the significant differences of FIGO stage (*p* = 0.005), LVI status (*p* = 0.003), CD3+ TILs in the central tumor area (*p* = 0.045), CD45RO+ TILs in the central tumor area (*p* = 0.033), CD45RO+ TILs in the invasive margin area (*p* = 0.033) were observed. As shown in Table 4, only the status of lymph node metastasis (*p* = 0.005) and tumor size (*p* = 0.002) were significantly different between patients with early stage cervical cancer and patients with locally advanced cervical cancer; however, no significant differences of tumor infiltrating immune cells were observed.

**Table 3:** Comparison of clinicopathological features between cervical cancer patients with different lymph node statuses.

Variable	*n*	Lymph node metastasis (−)	Lymph node metastasis (+)	*P* value
***FIGO stage***	96			0.005^**^
<IBII	47	45	2	
≥IBII	49	37	12	
**Histological type**	96			0.865
Squamous cell carcinoma	88	75	13	
adenocarcinoma	3	2	1	
Adenosquamous carcinoma	3	3	0	
Neuroendocrine small cell carcinoma	2	2	0	
**LVI status**	96			0.003^**^
−	61	57	4	
+	35	25	10	
**Parametrial invasion**	96			0.152
+	94	81	13	
+	2	1	1	
**Surgical margin status**	96			0.350
+	93	80	13	
+	3	2	1	
**Stromal invasion**	96			0.089
Superficial 1/3	36	35	1	
Middle 1/3	15	12	3	
Deep 1/3	45	35	10	
**Growth pattern**	96			0.336
Exophytic nodular	65	53	12	
Ulcerated nodular	5	4	1	
Endophytic nodular	21	20	1	
flat	5	5	0	
***Tumor size***	96			0.148
No visible lesion	5	5	0	
**<2**	17	17	0	
**≥2, <4**	59	47	12	
**≥4**	15	13	2	
***HPV status***	81			0.707
*Negative*	17	15	2	
*HPV-16* (+*)*	47	38	9	
*HPV-18* (+*)*	4	4	0	
*Other Hr HPV* (+*)*	13	12	1	
***Age (median(range))***	96	47.20 ± 9.94	46.93 ± 8.33	0.823
***Ki67 index(median(range))***	96	0.80(0.10–0.95)	0.775(0.20–0.90)	0.601
***CD3***_***IM***_***(median(range))***	96	200(40–500)	215(120–300)	0.684
***CD3***_***CT***_ ***(median(range))***	96	40(0–400)	20(0–80)	0.045^*^
***CD45RO***_***IM***_ ***(median(range))***	96	205(60–500)	170(100–250)	0.028^*^
***CD45RO***_***CT***_ ***(median(range))***	96	50(0–300)	10(0–120)	0.033^*^
***CD4***_***IM***_ ***(median(range))***	96	155(50–400)	190(100–250)	0.839
***CD4***_***CT***_ ***(median(range))***	96	17.5(0–180)	0(0–90)	0.099
***CD8***_***IM***_ ***(median(range))***	96	150(30–400)	130(80–200)	0.239
***CD8***_***CT***_ ***(median(range))***	96	50(0–400)	10(0–150)	0.050
***FOXP3***_***IM***_ ***(median(range))***	96	100(0–350)	90(0–250)	0.655
***FOXP3***_***CT***_ ***(median(range))***	96	0(0–150)	0(0–40)	0.338
***CD68***_***IM***_ ***(median(range))***	96	100(0–210)	115(5–200)	0.531
***CD68***_***CT***_ ***(median(range))***	96	40(0–200)	25(0–90)	0.130
***CD163***_***IM***_ ***(median(range))***	96	100(0–220)	100(30–50)	0.942
***CD163***_***CT***_ ***(median(range))***	96	100(0–220)	100(30–150)	0.180
***PD-L1 expression***	96			0.328
−	45	36	9	
+	23	21	2	
+ +	19	18	1	
+ + +	9	7	2	

**Notes.**

* *P* < 0.05 ** *P* < 0.01

**Table 4:** Comparison of clinicopathological features between cervical patients with different FIGO stages.

Variable	n	FIGO stage <IBII	FIGO stage ≥IBII	*P* value
**Histological type**	96			0.554
Squamous cell carcinoma	88	45	43	
adenocarcinoma	3	1	2	
Adenosquamous carcinoma	3	1	2	
Neuroendocrine small cell carcinoma	2	0	2	
**LVI status**	96			0.630
−	61	31	30	
+	35	16	19	
**Lymph node metastasis**	96			0.005^**^
−	82	45	37	
+	14	2	12	
**Parametrial invasion**	96			0.976
−	94	46	48	
+	2	1	1	
**Surgical margin status**	96			0.085
−	93	47	46	
+	3	0	3	
**Stromal invasion**	96			0.449
Superficial 1/3	36	19	17	
Middle 1/3	15	9	6	
Deep 1/3	45	26	19	
**Growth pattern**	96			0.059
Exophytic nodular	35	27	38	
Ulcerated nodular	5	3	2	
Endophytic nodular	21	12	9	
flat	5	5	0	
***Tumors size***	96			0.002^**^
No visible lesion	5	5	0	
<2	17	13	4	
≥2, <4	59	25	34	
≥4				
***HPV status***	81			0.176
***Negative***	17	5	12	
***HPV-16* (+*)***	47	28	19	
***HPV-18* (+*)***	4	1	3	
***Other Hr HPV* (+*)***	13	5	8	
***Age (median(range))***	96	47(24–71)	48(24–64)	0.950
***Ki67 index*** ***(median(range))***	96	0.80 (0.10–0.90)	0.80 (0.10–0.95)	0.948
***CD3***_***IM***_***(median(range))***	96	200(50–500)	200(40–400)	0.752
**CD3**_**CT**_ ***(median(range))***	96	40(0–400)	40(0–200)	0.632
**CD45RO**_***IM***_ ***(median(range))***	96	200(60–500)	220(100–450)	0.330
**CD45RO**_**CT**_ ***(median(range))***	96	40(0–300)	40(0–250)	0.848
***CD4***_***IM***_ ***(median(range))***	96	160(70–400)	160(50–380)	0.721
**CD4**_**CT**_ ***(median(range))***	96	10(0–120)	10(0–180)	0.847
**CD8**_***IM***_ ***(median(range))***	96	150(30–400)	150(30–380)	0.986
**CD8**_**CT**_ ***(median(range))***	96	50(0–400)	20(0–200)	0.845
***FOXP3***_***IM***_ ***(median(range))***	96	100(30–300)	100(0–350)	0.360
**FOXP3**_**CT**_ ***(median(range))***	96	0(0–150)	5(0–70)	0.392
**CD68**_***IM***_ ***(median(range))***	96	100(0–200)	110(0–210)	0.180
***CD68***_**CT**_ ***(median(range))***	96	30(0–200)	30(0–200)	0.930
**CD163**_***IM***_ ***(median(range))***	96	100(0–200)	100(20–220)	0.079
**CD163**_**CT**_ ***(median(range))***	96	40(0–200)	30(0–200)	0.877
***PD-L1 expression***	96			0.615
−	45	22	23	
+	23	13	10	
+ +	19	7	12	
+ + +	9	5	4	

**Notes.**

* *P* < 0.05 ** *P* < 0.01

The differences of clinicopathological features in patients with low-, intermediate-, and high risks were also investigated, as shown in Table 5, significant differences of status of lymph node (*p* = 0.000), LVI status (*p* = 0.000), stromal invasion status (*p* = 0.000), parametrial invasion status (*p* = 0.012), tumor size (*p* = 0.000) and tumor growth pattern (*p* = 0.015) among 3 groups of patients were observed. However, only CD45RO+ TILs in the invasive margin area (*p* = 0.018) and FOXP3+ TILs in the central tumor area (*p* = 0.009) were statistically different among patients with low-, intermediate-, and high risks in this study.

**Table 5:** Comparison of clinicopathological features between cervical patients with different risk profiles.

Variable	*n*	Low risk	intermediate risk	High risk	*P* value
***FIGO stage***	96				0.018^*^
<IBII	47	34	9	4	
≥IBII	49	23	12	14	
**Histological type**	96				0.937
Squamous cell carcinoma	88	54	18	16	
adenocarcinoma	3	1	1	1	
Adenosquamous carcinoma	3	1	1	1	
Neuroendocrine small cell carcinoma	2	1	1	0	
**LVI status**	96			T	0.000^**^
−	61	51	6	4	
+	35	6	15	14	
**Lymph node metastasis**	96				0.000^**^
−	82	57	21	4	
+	14	0	0	14	
**Parametrial invasion**	96				0.012^*^
−	94	57	21	16	
+	2	0	0	2	
**Surgical margin status**	96				0.090
−	93	56	21	16	
+	3	1	0	2	
**Stromal invasion**	96				0.000^**^
Superficial 1/3	36	35	0	1	
Middle 1/3	15	7	4	4	
Deep 1/3	45	15	17	13	
**Growth pattern**	96				0.015
Exophytic nodular	65	38	11	16	
Endophytic nodular	21	10	10	1	
Ulcerated nodular	5	4	0	1	
flat	5	5	0	0	
***Tumor size***	96				0.000^**^
No visible lesion	5	5	0	0	
<2	17	16	1	0	
≥2, <4	59	36	8	15	
≥4	15	0	12	3	
***HPV status***	81				0.544
*Negative*	17	8	6	3	
*HPV-16* (+*)*	47	26	11	10	
*HPV-18* (+*)*	4	2	1	1	
*Other Hr HPV* (+*)*	13	11	0	2	
***Age (median(range))***	96	48(24–71)	47(27–61)	48.5(29–63)	0.655
***Ki67 index*** ***(median(range))***	96	0.80 (0.10–0.95)	0.75 (0.10–0.85)	0.775 (0.20–0.90)	0.658
***CD3***_***IM***_***(median(range))***	96	220(50–500)	200(40–400)	200(120–300)	0.318
**CD3**_**CT**_ ***(median(range))***	96	50(0–400)	30(0–200)	20(0–80)	0.137
**CD45RO**_***IM***_ ***(median(range))***	96	220(60–500)	220(100–350)	170(100–250)	0.018^*^
**CD45RO**_**CT**_ ***(median(range))***	96	40(0–300)	50(0–150)	20(0–120)	0.150
***CD4***_***IM***_ ***(median(range))***	96	160(50–400)	160(120–350)	170(100–250)	0.856
**CD4**_**CT**_ ***(median(range))***	96	20(0–180)	10(0–120)	0(0–90)	0.088
**CD8**_***IM***_ ***(median(range))***	96	150(30–400)	150(70–320)	130(80–200)	0.400
**CD8**_**CT**_ ***(median(range))***	96	50(0–400)	30(0–200)	10(0–150)	0.119
***FOXP3***_***IM***_ ***(median(range))***	96	100(0–350)	100(0–220)	90(0–250)	0.941
**FOXP3**_**CT**_ ***(median(range))***	96	10(0–150)	0(0–50)	0(0–40)	0.009^**^
**CD68**_***IM***_ ***(median(range))***	96	100(0–210)	120(0–200)	115(5–200)	0.130
***CD68***_**CT**_ ***(median(range))***	96	30(0–200)	50(0–110)	30(0–100)	0.448
**CD163**_***IM***_ ***(median(range))***	96	100(0–220)	100(0–220)	110(30–150)	0.513
**CD163**_**CT**_ ***(median(range))***	96	40(0–200)	40(0–160)	20(0–110)	0.471
***PD-L1 expression***	96				0.761
−	45	24	10	11	
+	23	15	6	2	
+ +	19	12	4	3	
+ + +	9	6	1	2	

**Notes.**

* *P* < 0.05 ** *P* < 0.01

Spearman’s rank correlation coefficient analysis was furtherly performed to evaluate the relationship between clinicopathologcial parameters and prognosis of cervical cancer patients after radical hysterectomy. According to Table 6, FIGO stage, LVI status, lymph node status, parametrial invasion status, stroma invasion status, and tumor size demonstrated positive correlation with risk stratification in a significant level (*P* = 0.005, 0.020, 0.000, 0.022, 0.000, and 0.000 respectively), while CD45RO+ TILs in the invasive margin area and FOXP3+ TILs in the central tumor area demonstrated negative correlation with risk stratification in a significant level (*P* = 0.031, 0.009 respectively). However, a further multiple logistic regression analysis did not identify any independent variables for estimation of risk stratification in our study (data not shown).

**Table 6:** Spearman’s rank correlation coefficient analysis between risk stratification and clinicopathological features.

Variables	n	Spearman correlation coefficient with risk group	*P* value
*FIGO stage*	96	0.283^**^	0.005
*Histopathologic type*	96	0.132	0.201
*HPV status*	81	−0.161	0.151
*Age*	96	−0.026	0.798
*Ki67 index*	96	−0.002	0.984
*LVI status*	96	0.614^**^	0.000
*Lymph node metastasis*	96	0.666^**^	0.000
*Parametrial invasion*	96	0.233^*^	0.022
*Surgical margin status*	96	0.144	0.163
*Stromal invasion status*	96	0.548^**^	0.000
*Growth pattern*	96	−0.121	0.241
*Tumor size*	91	0.468^**^	0.000
*PD-L1 expression*	96	−0.112	0.276
old*CD3*_***IM***_	96	−0.154	0.133
*CD3*_***CT***_	96	−0.187	0.068
*CD45RO*_***IM***_	96	−0.221^*^	0.031
*CD45RO*_***CT***_	96	−0.185	0.071
*CD4*_***IM***_	96	−0.024	0.814
*CD4*_***CT***_	96	−0.178	0.083
*CD8*_***IM***_	96	−0.122	0.236
*CD8*_***CT***_	96	−0.190	0.064
*FOXP3*_***IM***_	96	0.003	0.977
*FOXP3*_***CT***_	96	−0.264^**^	0.009
*CD68*_***IM***_	96	0.186	0.070
*CD68*_***CT***_	96	−0.066	0.525
*CD163*_***IM***_	96	0.118	0.251
*CD163*_***CT***_	96	−0.124	0.227

**Notes.**

* *P* < 0.05 ** *P* < 0.01

## Discussion

The tumor microenvironment is a niche that supports tumor development and progression, which ultimately affects response to therapy and clinical outcome (Catalano et al., 2013; Fridman et al., 2012; Gajewski, Schreiber & Fu, 2013; Taube et al., 2018). Utilization of the infiltrating immune cells as prognostic biomarkers has been reported in both solid and hematological malignant diseases (Becht et al., 2016; Burugu, Asleh-Aburaya & Nielsen, 2017; Cho et al., 2017; Pages et al., 2018; Shi et al., 2018; Taube et al., 2018; Teng et al., 2015; Zikich, Schachter & Besser, 2016). Tumor infiltrating T cells (TILs) and tumor associated macrophages (TAMs) are key components of cellular immune response in tumor microenvironment (Becht et al., 2016; Galdiero et al., 2013; Reiser & Banerjee, 2016), in addition, PD-1/PD-L1 is an important immune checkpoint pathway in mediating tumor cell evasion from immune surveillance, and immunotherapies targeting PD-1/PD-L1 signaling pathway has demonstrated great efficacy in multiple type of cancers (FDA, 2019; Le et al., 2015; Sharma & Allison, 2015). However, the clinical value of the inflammatory tumor microenvironment components in cervical cancer patients with radical hysterectomy was still elusive. In our study, the potential link between tumor infiltrating immune cells (including TILs, TAMs, etc), PD-L1 expression of tumor cells and risk stratification of cervical cancer patients with radical hysterectomy was investigated, and significant differences of distribution of CD3+, CD45RO+, CD4+, CD8+ and FOXP3+ TILs were found to be associated with cinicopathological features. These results was partly consistent with previous related studies (Luo et al., 2015; Piersma et al., 2007); however, our study evaluated the influence of both TILs and TAMs to the risk stratification for cervical cancer patients and only subsets of TILs was proved to be potential prognostic biomarkers.

Cervical cancer is still a major health concern with high morbidity and mortality in women worldwide. Radical hysterectomy has been widely used as the standard treatment for early resectable cervical cancer patients (Cohen et al., 2019; Torre et al., 2017). Several clincopathological variables have been recognized as risk factors, such as FIGO stages, LVI status, lymph node metastasis, stromal invasion, parametrial invasion, tumor size, etc (Cohen et al., 2019; Delgado et al., 1990; Halle et al., 2017). Risk stratification of cervical cancer is widely used in determining treatment strategies and predicting prognosis in patients with invasive cervical cancer who took radical hysterectomy. Risk stratification of cervical cancer is based on adverse pathologic factors such as positive pelvic nodes, parametrial infiltration, positive margins, and deep stromal invasion. Cervical cancer patients can be categorized into three groups: high-risk, intermediate-risk, and low-risk groups (Bhatla et al., 2018). Clinically, different treatment strategy should be given to cervical cancer patients with different risk stratification. In the present study, the widely used risk factors including FIGO stage, LVI status, lymph node status, parametrial invasion status, stroma invasion status, and tumor size were found to be positively correlated with risk stratification of cervical cancer. In addition, we found negative correlation between subsets of TILs (CD45RO+ TILs in the invasive margin area and FOXP3+ TILs in the central tumor area) and risk stratification, which confirmed the inherent association between inflammatory/immune tumor microenvironment and clinical outcome.

The current study found that the densities of CD3+ TILs in the tumor center area are significant higher in both LVI (+) patients and patients with lymph node metastasis, however, no similar results were identified in patients with different FIGO stages. This discrepancy might be attributed to the inaccuracy of the present FIGO staging system for cervical cancer, which was determined solely on clinical data. However, in 2018, a new version of FIGO staging system for cervical cancer was introduced to allow available imaging and pathological findings (including lymph node status) to assign the stage (Bhatla et al., 2019), and the improved accuracy would be more helpful for clinical practice and research.

Tumor infiltrating regulatory T cells (Tregs) are reported to extensively exist in most cancers participating in inhibition of immune responses, tumor metastasis, tumor recurrence, and treatment resistance (Curiel et al., 2004; Lee et al., 2008; Li et al., 2016; Saito, Nishikawa & Wada, 2016). FOXP3 is a pivotal nuclear transcription factor and useful biomarker of Tregs (Shou et al., 2016). In our study, the density of FOXP3+ TILs in the central tumor area demonstrated negative correlation with risk stratification in a significant level (*P* = 0.009), which suggest the importance of Tregs as both prognostic biomarkers and therapeutic targets for cervical cancer patients.

In recent years, immune score system based on quantification of CD3+ and cytotoxic CD8+ T cells densities in the tumor and in the invasive margin has been proved to be a robust prognostic biomarker for colon cancer and other solid cancers (Hendry et al., 2017a; Hendry et al., 2017b; Pages et al., 2018). However, few studies on the utilization of immune scores of cervical cancer have been reported. Since not all patients with cervical cancer are suitable for radical hysterectomy, the validated immune score system can be introduced to evaluate the small biopsy samples of patients with cervical cancer for estimating risk stratification and prognosis. Our study demonstrated only two subsets of TILs negatively associated with risk stratification in a significant level (*P* = 0.031, 0.009 respectively), which suggested the possible clinical value of scoring TILs in the microenvironment of cervical cancer tissues in pathology laboratories, however, this hypothesis required further investigations for validation.

In this study, the age, Ki67 index and distribution of tumor infiltrating CD68+ and CD163+ TAMs in cervical cancer tissue were also investigated, however, no significant difference of age, Ki67 index, CD68+ or CD163+ TAMs in the tumor microenvironment was identified between patients correlated with LVI status, lymph node metastasis and FIGO stage, and further analysis failed to find any correlations between TAMs and risk stratification in cervical cancer patients. As to PD-1/PD-L1 signaling pathway, no significant correlation was identified in our study, however, the negative results might attributed to the limited numbers of cases and subjectivity in both immunohistochemistry technique and evaluation procedures.

The retrospective nature, biases including tissue fixation, immunostain and its evaluation, and the sample size represent relevant limitations in this study. Moreover, the insufficient survival data because of the difficulties in follow-up for cervical cancer patients of this area hampered the necessary survival analysis. Nevertheless, our study presents the first report on the correlation between cervical cancer risk stratification and clinicopathological features including inflammatory/immune tumor microenvironment factors.

In conclusion, our work suggested that assessment of CD45RO+ TILs in the invasive margin area and FOXP3+ TILs in the central tumor area of cervical cancer tissue might be helpful for choosing therapeutic strategies and prognostication for cervical cancer with radical hysterectomy. However, large cohort studies of cervical cancer patients with complete follow up are needed to further examine the robustness and validity of these biomarkers before introduced to pathological laboratories.

##  Supplemental Information

10.7717/peerj.7804/supp-1 Supplemental Information 1 Clinico-pathological data of cervical cancer

## Additional Information and Declarations

### Data Availability

The following information was supplied regarding data availability: The raw measurements are available in the Supplemental File.
